# Biochef: a client-side WebAssembly-based workflow builder for genomic data analysis

**DOI:** 10.1186/s12859-026-06431-1

**Published:** 2026-04-09

**Authors:** Joaquim Rosa, João Andrade, Jorge Miguel Silva, José Luis Oliveira

**Affiliations:** IEETA/DETI, LASI, University of Aveiro, Campus Universitário de Santiago, Aveiro, 3810-193 Portugal

**Keywords:** WebAssembly, Bioinformatics workflows, Web-based applications, Workflow management systems, Human-computer interaction, Genomic data processing

## Abstract

**Background:**

Genomics analyses often rely on command-line tools executed via remote servers, imposing usability barriers for non-technical users and raising privacy concerns. WebAssembly (WASM) enables native-code execution directly in web browsers, eliminating installations and data transfers.

**Results:**

We introduce BioChef, a client-side genomic workflow platform that uses WASM. BioChef compiles a genomics toolkit into browser-executable modules and exposes them through a drag-and-drop GUI designed to be intuitive. The system provides real-time validation, flexible input methods (form-based and JSON), intermediate step inspections, and reproducible workflows exportable as bash scripts or configuration files. Performance benchmarks across major browsers (Chromium, Gecko, WebKit) demonstrate rapid initialization (LCP 0.583 s), responsive interactivity (INP 30.5 ms), minimal layout shifts (CLS 0.01), and acceptable overhead (average 181.5 ms initial WASM module load). Although browser execution introduced performance penalties ($\sim$130$\times$ slower than native), BioChef workflows still significantly outperformed traditional web services such as Galaxy by avoiding network delays and server-side queueing (11.3$\times$ faster in a standard pipeline benchmark).

**Conclusions:**

BioChef shows how WebAssembly on the client side can democratize genomic data processing, ensuring privacy, reproducibility and ease of use without external dependencies. To our knowledge, this is the first fully client-side, graphical genomic workflow environment powered by WASM.

## Introduction

Modern biological research relies heavily on computational tools to process, analyze, and manipulate genomic sequence data [1, 2]. These essential bioinformatics applications handle critical tasks such as DNA sequence manipulation, file format conversion between standards like FASTA and FASTQ, genomic complement operations, and various analytical procedures that form the backbone of contemporary genomics workflows. However, the vast majority of these genomic analysis tools are distributed predominantly as command-line applications, especially on Unix-like systems [3], creating a significant accessibility barrier for researchers in health sciences and biology. While these tools offer capabilities for sequence manipulation, format conversion, and genomic operations, their utilization requires technical expertise in compilation, installation, and terminal-based operations that extends well beyond the domain knowledge of most biological researchers [4, 5].

This technical barrier presents a substantial obstacle for researchers in the health sciences and biology domains, who possess deep domain knowledge, but may lack the computational skills necessary to take advantage of these genomic tools [6, 7]. Although informatics professionals typically have the necessary technical background to compile, install, and execute command-line tools, healthcare professionals and biologists often find themselves excluded from directly utilizing these resources. This digital divide limits the accessibility of cutting-edge genomic analysis capabilities and creates dependencies on technical intermediaries, potentially slowing research progress and reducing the autonomy of domain experts [8, 9].

Beyond accessibility challenges, server-side implementations of bioinformatics platforms introduce significant security and privacy concerns when handling sensitive genomic data [10]. Server-based solutions require researchers to upload sensitive data to external systems, creating vulnerabilities during transmission and storage while raising compliance issues with regulatory frameworks such as HIPAA and GDPR [11]. The centralized nature of server infrastructure presents attractive targets for cyber attacks, and multi-tenancy architectures risk data exposure between research groups [12]. These security concerns are particularly acute in clinical genomics, where patient privacy is paramount and data breaches can have severe consequences.

Traditional approaches to address this challenge have included server-based platforms such as Galaxy [13, 14], which provide web interfaces for bioinformatics workflows. Although these solutions have proven valuable, they require back-end infrastructure, which again raise concerns about data privacy when handling sensitive genomic information, and introduce latency through client–server communication. Recent advances in web technologies, particularly WebAssembly (WASM) [15], have opened new possibilities for client-side execution of compiled applications directly within web browsers [16, 17]. The Emscripten compiler toolchain [18] enables the compilation of existing C/C++ bioinformatics applications to WebAssembly, making it possible to execute these tools directly in web browsers without modification of the original source code.

We present BioChef, a client-side web application that addresses these limitations by providing a browser-based interface, designed to be intuitive, for genomic tool execution and workflow construction. BioChef leverages the Genomics-Proteomics Toolkit (GTO) [19], a comprehensive collection of C-based tools for genomic sequence analysis and manipulation, and compiles them into WebAssembly using Emscripten for browser execution. This approach eliminates the traditional prerequisites of C compilation knowledge and terminal proficiency by presenting these tools through a user-friendly graphical interface built in ReactJS [20] and MaterialUI [21]. The application operates entirely within the user’s browser, ensuring data privacy, eliminating installation requirements, and providing immediate access to a comprehensive suite of genomic manipulation tools.

The key contributions of this work include: (1) a novel client-side approach to genomic workflow construction using WebAssembly-compiled tools, (2) a drag-and-drop interface that makes complex bioinformatics operations accessible to non-technical users, (3) a comprehensive implementation encompassing sequence manipulation, format conversion, and genomic analysis operations, and (4) a demonstration that browser-based bioinformatics can achieve practical performance for real-world genomic datasets.

This paper describes the implementation and evaluation of BioChef, demonstrating how modern web technologies can facilitate access to genomic analysis tools and workflow building while achieving practical performance for typical genomic datasets within the constraints of browser-based execution.

## Background

### WebAssembly technology

WebAssembly (WASM) is a binary instruction format designed as a portable compilation target for high-level languages, enabling deployment on the web for client and server applications [22]. Originally developed to overcome the performance limitations of JavaScript in web browsers, WebAssembly provides near-native execution speed while maintaining the security and portability characteristics essential for web applications.

WebAssembly offers significant advantages for scientific computing applications through its performance characteristics. While small scientific kernels can achieve execution speeds within 10–20% of native code, larger applications typically experience greater overhead, with recent comprehensive evaluations showing average performance penalties of 45–55% compared to native implementations [23]. Nevertheless, WebAssembly significantly outperforms JavaScript implementations across all application categories. The technology’s language-agnostic design enables existing C/C++ codebases to be compiled to WebAssembly with minimal modifications, making it particularly valuable for porting established scientific software to web environments. WebAssembly modules execute within a sandboxed environment that provides memory safety and prevents unauthorized system access, addressing security concerns inherent in web-based scientific computing. Additionally, the platform-independent nature of WebAssembly ensures consistent execution across different operating systems and hardware architectures through standardized browser runtimes.

#### Emscripten compilation toolchain

Emscripten serves as the primary compilation toolchain for translating C and C++ code to WebAssembly [24]. As an LLVM-based compiler, Emscripten provides a complete development environment that encompasses both compilation capabilities and a comprehensive runtime system designed to emulate POSIX APIs within the constrained browser environment. This emulation layer enables traditional command-line applications to function within web browsers by providing familiar file system operations, memory management routines, and standard library functions.

The Emscripten toolchain generates low-level JavaScript bindings that facilitate WebAssembly module loading and provide basic function access interfaces. Through aggressive optimization strategies including dead code elimination and function inlining, Emscripten minimizes WebAssembly module size while maintaining performance characteristics [25]. The compilation system offers flexible runtime configuration options, supporting dynamic memory growth and custom function export specifications to accommodate diverse application requirements.

### Genomics-proteomics toolkit (GTO)

The Genomics-proteomics toolkit (GTO) is a comprehensive suite of command-line tools designed for genomic and proteomic data analysis [19]. GTO provides a modular, UNIX-pipe compatible toolkit that supports multiple file formats including FASTA, FASTQ, and raw sequence data. The majority of this toolkit implements a “LEGO-like” philosophy, enabling users to construct complex analytical pipelines by combining individual tools through standard input/output streams.

The GTO tools provide comprehensive genomic data processing capabilities spanning sequence manipulation, format conversion, and analytical operations. These tools handle fundamental genomic tasks including sequence extraction, complement and reverse operations, quality filtering, and mutation simulation across FASTA and FASTQ formats. The toolkit supports essential format conversions between different genomic file types and enables translation between DNA and amino acid sequences. Advanced functionality includes quality score analysis, pattern-based sequence extraction, read splitting and merging operations, and statistical analysis of genomic data properties. Additional utilities provide mathematical operations on numerical data, text processing capabilities, and information extraction functions that facilitate both basic sequence manipulation tasks and complex analytical workflows in genomic research.

The toolkit’s design philosophy emphasizes modularity and interoperability, with each tool accepting standard input streams and producing compatible outputs for downstream processing. This architecture enables construction of complex analytical workflows through simple command-line piping, making it particularly suitable for both automated processing and interactive analysis scenarios.

## Related work

The landscape of bioinformatics workflow management and genomic data analysis has evolved significantly with the emergence of web-based platforms and client-side computing technologies. This evolution has been driven by the need to address key challenges in genomic analysis: computational accessibility, data privacy concerns, and the growing complexity of bioinformatics workflows. Modern approaches span from traditional server-based systems that leverage substantial computational resources to innovative client-side platforms that execute directly in web browsers, each offering distinct advantages and addressing different user needs.

Recent advances in WebAssembly technology have enabled the development of client-side bioinformatics applications that execute directly in web browsers without requiring server infrastructure. ViralWasm [16] exemplifies this approach, representing a client-side web application suite specifically designed for viral genomics analysis. The platform utilizes WebAssembly to execute original command-line tools directly in the browser through the biowasm repository of pre-compiled tools [26], supporting both individual tool execution and complete analysis pipelines. ViralWasm-Consensus provides consensus sequence generation from FASTQ, SAM, or BAM files, while ViralWasm-Epi offers molecular epidemiology analysis capabilities. The platform achieves execution speeds within 2–3 of native command-line counterparts while maintaining complete data privacy through client-side processing. Similarly, kana [17] demonstrates the potential of WebAssembly for single-cell genomics data analysis entirely within the browser environment. The platform leverages WebAssembly-compiled C/C++ libraries to perform computationally intensive single-cell analysis workflows, including quality control, normalization, dimensionality reduction, and clustering. Kana can analyze datasets containing more than 100,000 cells in minutes on typical desktop computers while offering a simplified one-click workflow interface for nontechnical users. Specialized WebAssembly applications have also emerged for specific domains within genomics. Chromatic [27] represents the first oncology bioinformatics tool built specifically with WebAssembly technology, serving as a cancer genome viewer that enables client-side processing of genomic data files for visual inspection of genomic variations. The tool allows researchers to visually inspect genomic variations identified through next-generation sequencing of cancer data sets to determine whether such calls are valid, providing access to public cancer data sets, and featuring easy-to-use interfaces designed for scientists without extensive bioinformatics expertise. Educational platforms like sandbox.bio [28] provide interactive browser-based tutorials for bioinformatics tools, using the biowasm ecosystem [26] to run C / C++ bioinformatics tools directly in the browser while simulating a Linux command line environment. fastq.bio [29] enables browser-based quality control of FASTQ sequencing files entirely on the client side, using a WebAssembly-compiled version of the fastp tool executed through the aioli framework and biowasm environment. These WebAssembly-based platforms collectively demonstrate the maturation of client-side computing for bioinformatics, offering installation-free access, enhanced data privacy, and near-native performance across diverse genomic analysis domains.

The client-side approach also extends to JavaScript-based solutions that predate widespread WebAssembly adoption. UBiT2 [30] offers a purely client-side web application for gene expression data analysis, focusing on RNA-sequencing and qPCR data processing. Although implemented in JavaScript rather than WebAssembly, UBiT2 provides offline installation-free analysis capabilities with interactive visualizations and supports standard analyses including quality control, hierarchical clustering, principal component analysis, and differential expression analysis. This shows that effective client-side bioinformatics tools can be developed using traditional web technologies when WebAssembly is not available or necessary.

Traditional server-based platforms continue to play a significant role in bioinformatic workflow management, offering tool collections and advanced workflow capabilities that leverage substantial computational resources. Galaxy [13] represents one of the most established workflow management systems in bioinformatics, providing an extensive collection of tools and genomic analysis capabilities through a web-based interface designed for biologists without programming experience. The platform supports both individual tool execution and visual workflow construction, enabling access to substantial computational resources, but requires careful consideration of data privacy when handling sensitive genomic information. BioDepot Workflow Builder [31] offers a containerized workflow construction platform that enables users to create and execute reproducible bioinformatics workflows using a drag-and-drop interface. The platform represents computational tasks as graphical widgets that correspond to Docker containers, allowing for modular workflow construction across different local and cloud platforms. Playbook Workflow Builder [32] provides a web-based platform for dynamic construction and execution of bioinformatic workflows through a growing network of semantically annotated API endpoints and data visualization tools, focusing on user-friendly interfaces for experimental biologists while maintaining integration capabilities with multiple data sources and analysis tools.

Earlier workflow management systems focused on distributed computing architectures and agent-based technologies. BioWMS [33] was a web-based workflow management system that allowed researchers to create bioinformatic workflows using a graphical drag-and-drop interface. Users could design workflows by connecting pre-built activities such as BLAST searches, sequence retrievals, and global alignments through visual control flow patterns including sequences, parallel execution, conditional branching, and loops. The platform included a library of domain-specific bioinformatics activities and supported standard workflow specification languages like XPDL, enabling workflows to be edited by other compliant applications.

Modern cloud-based platforms address the computational demands of large-scale genomic analysis while providing user-friendly interfaces and collaborative capabilities. Closha 2.0 [34] represents a cloud-based genomics platform featuring drag-and-drop workflow construction capabilities with a no-code interface for researchers with limited programming experience, while also providing integrated Python and R scripting support for advanced users who require custom analysis components. The platform utilizes existing containerized tools to provide genomic analysis capabilities through cloud infrastructure, demonstrating how cloud computing can make bioinformatics analyses accessible to broader research communities.

The diversity of approaches represented by these platforms demonstrates the evolving landscape of bioinformatic tool accessibility. Client-side implementations prioritize data privacy and installation-free usage, while server and cloud-based platforms provide extensive computational resources and collaborative capabilities. Each approach addresses different aspects of the fundamental challenges in genomic workflow management.

In the following sections, we detail BioChef’s implementation architecture, demonstrate its interface functionality and performance characteristics, and provide a comparative analysis with the platforms reviewed in this section.

## Implementation

BioChef is a client-side web application that uses ReactJS as the primary front-end framework, with MaterialUI providing the component library for user interface elements. The application architecture follows a modular design pattern that organizes functionality into specialized components that handle distinct aspects of the execution of genomic tools and workflow management. This section details the component architecture and implementation strategies employed to achieve the platform’s dual functionality of individual tool testing and visual workflow construction.

### System architecture overview

**Fig. 1:**
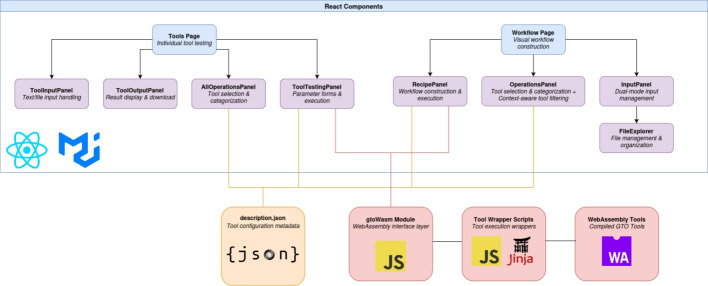
BioChef application architecture showing the ReactJS component layer and underlying infrastructure modules. The ToolsPage provides individual tool testing capabilities, while the WorkflowPage enables visual workflow construction and execution. The system integrates description.json for tool configuration, gtoWasm for WebAssembly interface management, dynamically generated wrapper scripts, and compiled WebAssembly tools for client-side genomic analysis

The system comprises two primary layers: the ReactJS application layer that provides user interaction capabilities, and the underlying WebAssembly infrastructure that enables client-side execution of genomic tools. The integration between these layers is facilitated by a tool configuration system and dynamically generated wrapper scripts (Fig. 1).

### Tool configuration system

The *description.json* file serves as the central configuration repository that drives the dynamic behavior of the BioChef platform. This metadata file contains detailed specifications for each genomic tool in the GTO toolkit, including input/output format requirements, parameter definitions with type specifications and validation constraints, and execution requirements. The configuration system supports various parameter types such as file-based inputs and numeric constraints, while accommodating multi-output tools that produce multiple distinct data streams. This metadata enables dynamic UI generation, real-time parameter validation, and automatic tool compatibility analysis for workflow construction.

### WebAssembly infrastructure

The client-side execution capabilities are enabled through a WebAssembly infrastructure that compiles C/C++ genomic tools using the Emscripten toolchain. The compilation process transforms GTO toolkit [19] source code into browser-executable modules while preserving command-line interfaces and managing shared dependencies. Dynamically generated wrapper scripts provide JavaScript interfaces that abstract WebAssembly complexity, handle parameter marshaling, and manage multi-output scenarios. The wrapper generation process uses Jinja2 templates [35] to create tool-specific JavaScript interfaces based on metadata from the configuration system. The gtoWasm module serves as the integration layer, implementing lazy loading strategies, resource management, and standardized execution interfaces between the ReactJS application and WebAssembly modules.

### ReactJS application architecture

The application is structured around two primary interfaces: the ToolsPage for individual tool testing and the WorkflowPage for visual workflow construction. Each interface comprises specialized components that collectively provide an environment for genomic data analysis.

#### ToolsPage components

The ToolsPage implements a three-panel architecture through specialized React components that separate tool discovery, parameter configuration, and result visualization concerns.

Tool discovery and organization are handled by the AllOperationsPanel component, which organizes the GTO toolkit into categorized sections with hierarchical navigation and search functionality. The component implements debounced search capabilities and manages tool selection state through React hooks, providing dynamic filtering of the tool collection based on user input.

The ToolTestingPanel component provides a dynamically generated form based on tool configuration metadata from the description.json file. This component integrates contextual help through help function execution of the selected tool, manages parameter validation state for tool execution, and coordinates the tool execution through the gtoWasm interface layer.

The testing environment is completed through the ToolInputPanel and ToolOutputPanel components, which handle data input modalities and result presentation respectively. The ToolInputPanel supports multiple input methods, including direct text entry, file uploads, and a collection of predefined small sample datasets, to be used in the tool execution, while the ToolOutputPanel provides result visualization with download functionality.

To facilitate onboarding, the ToolsPage includes an interactive guided tour implemented using the Driver.js library, which provides step-by-step explanations of the main interface components and their functionality (Supplementary Figure 21). There also exists a button that allows users to restart the tour at any time.

#### WorkflowPage components

The WorkflowPage implements workflow construction through a multi-component architecture, shown on the right side of the React Components section (Fig. 1), that supports drag-and-drop pipeline creation and real-time execution feedback.

At the core of the workflow construction interface is the *RecipePanel* component, which manages the complete workflow lifecycle through React state management patterns and real-time execution capabilities. A critical data structure within this component is the output mapping system, which associates each tool ID in the workflow with its corresponding output(s). This mapping supports the complete validation logic for workflow steps, ensuring that inputs and outputs between tools are compatible while enabling users to visualize intermediate results throughout the workflow execution. This component integrates the @dnd-kit library [36] for drag-and-drop functionality, enabling users to reorganize tool components within the workflow as needed, and if possible. The component supports dual input modes (CLI and File Explorer Modes) inherited from the InputPanel component, allowing the preservation of multiple results when receiving multiple inputs simultaneously. Additionally, it handles the passage of multiple outputs between workflow steps when tools generate more than one output file. The component manages mid-workflow tool insertion through the dynamic compatibility analysis previously mentioned. And finally, it provides workflow persistence and sharing mechanisms through both JSON configuration export for later import and executable script generation for local workflow execution.

The OperationsPanel component implements intelligent tool filtering based on current workflow context, analyzing data type compatibility and workflow state to present contextually appropriate tool selections. Unlike the comprehensive tool listing in AllOperationsPanel, this component dynamically adapts available operations based on workflow progression.

Data input management is handled through the InputPanel component, which provides a tabbed interface supporting both direct text entry and hierarchical file management. The component implements data type detection for both input types through pattern matching algorithms, to make the OperationsPanel update instantly with the matching tools for that type. The InputPanel incorporates the FileExplorer component for hierarchical file organization. It provides file upload capabilities with size limiting and validation, to ensure that the user don’t crash it’s own browser or uploads files that don’t match the supported by the platform. It also implements file system operations including folder/ZIP upload with structure preservation, ZIP archive extraction right after upload, file information visualization and file/folder delete.

Like the ToolsPage, the WorkflowPage incorporates an interactive guided tour to assist users in understanding workflow construction (Supplementary Figure 22). The interface also provides an option to import an example recipe, enabling users to explore a preconfigured workflow as a starting point for learning or experimentation, with the tour being able to be restarted at any time through a button.

### Performance optimization

The implementation includes several performance optimization strategies to ensure responsive user interaction despite the computational complexity of genomic data processing. Debounced input handling prevents excessive validation cycles during user input. Lazy loading of WebAssembly modules reduces initial application load time while maintaining rapid tool execution. Local storage integration provides session persistence without requiring server-side state management. The output mapping system further contributes to performance by supporting incremental workflow execution, processing only affected workflow segments when modifications occur. Also, for scenarios with multiple inputs, the only output map entry that is updated is the one being shown on screen, the batch processing is only done when the user automatically saves the results. Additionally, for scenarios involving multiple inputs, the system optimizes performance by updating only the output map entry currently displayed to the user, while batch processing for result saving occurs only when users explicitly initiate the save operation.

## Results

### Tool testing interface

The ToolsPage interface provides an environment for the exploration and testing of individual genomic tools, organized through a three-panel layout that facilitates tool discovery, parameter configuration, and result visualization.

**Fig. 2:**
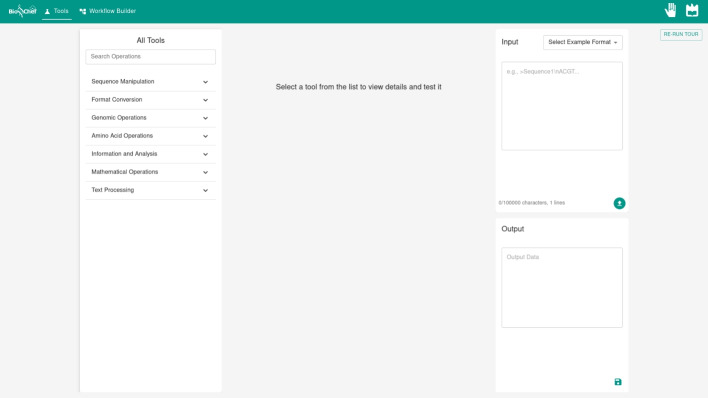
Initial interface state showing tool selection panel with categorized genomic operations and empty input/output panels

The initial interface state presents users with a tool selection panel on the left side, displaying the complete collection of available genomic operations organized by functional categories (Fig. 2). The tool selection panel implements a hierarchical organization structure that enables users to navigate through sequence manipulation, format conversion, genomic operations, amino acid operations, information and analysis, mathematical operations, and text processing categories.

Each tool entry provides immediate access to descriptive information through hover interactions, as demonstrated with the fasta_extract tool selection (Supplementary Fig. 1), where contextual help displays the tool’s primary function: “Extracts sequences from a FASTA file” with clear input and output format specifications.

Upon tool selection, the central tool testing panel dynamically renders the tool-specific interface, presenting supported input/output formats and available parameters (Supplementary Fig. 2). The interface distinguishes between required and optional parameters through visual indicators, with the fasta_extract tool example showing optional flags for sequence initialization (init) and termination (end) positions.

The input management system provides three distinct data entry modalities to accommodate different user workflows and data sources. Users can utilize pre-configured example data samples that demonstrate proper input formatting for each tool, manually enter custom sequence data directly into the input text area, or upload files from their local filesystem. The example demonstrates the selection of a sample FASTA sequence, providing users with immediate access to functional test data without requiring external file preparation (Supplementary Fig. 3).

The execution interface provides an intuitive and clear “RUN TOOL” button, which when clicked, initiates the WebAssembly-based tool processing (Supplementary Fig. 4).

Upon execution completion, the output panel displays the processed results in a read-only format that preserves output formatting while providing integrated download capabilities (Supplementary Fig. 5). The example output demonstrates the fasta_extract tool successfully extracting the first 20 nucleotides from the input sequence, showing the transformed sequence “TTGCACTGACCTGAAGTCTT” while maintaining the original FASTA header format.

### Workflow construction interface

The WorkflowPage provides a environment for visual workflow construction and execution, implementing a three-panel architecture that supports complex genomic analysis pipeline development through a drag-and-drop interface designed to be intuitive.

**Fig. 3:**
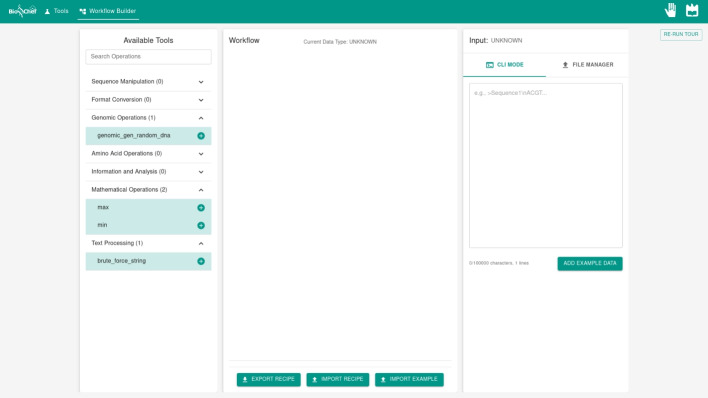
Initial state of the workflow construction interface showing the three-panel layout: Available Tools panel (left), Workflow panel (center), and Input panel (right)

The initial interface state presents a clean, organized workspace designed to facilitate intuitive workflow development (Fig. 3). The left panel displays the “Available Tools” section, which organizes the complete collection of genomic operations into expandable categories including Sequence Manipulation, Format Conversion, Genomic Operations, Amino Acid Operations, Information and Analysis, Mathematical Operations, and Text Processing. Each category displays the number of available tools in parentheses, enabling users to quickly assess the scope of operations within each functional domain.

The central “Workflow” panel serves as the primary construction area, initially displaying an empty workspace with “Current Data Type: UNKNOWN” status indicator, which will be updated to reflect the last workflow tool output type each time the workflow changes. This panel is designed to accommodate the visual representation of workflow steps, intermediate results, and data flow connections between operations. At the bottom of this panel, “EXPORT RECIPE” and “IMPORT RECIPE” buttons provide workflow persistence capabilities, enabling users to save constructed workflows as configuration files or executable scripts, and restore previously developed workflows for modification or reuse. The “IMPORT EXAMPLE“ button, also present there, allows users to import a ready-made example workflow and use it as a starting point for testing the workflow features.

The right panel implements the “Input” management system with dual-mode functionality indicated by “CLI MODE” and “FILE MANAGER” tabs. The Input panel header displays “UNKNOWN” status, which dynamically updates to reflect the detected data type as users provide input data. In CLI MODE, an ADD EXAMPLE DATA button is available, allowing for quickly populating the input field with sample data for testing.

#### Input management system

The workflow construction interface provides two distinct input modalities designed to accommodate different user workflows and data processing requirements: CLI Mode for direct text input and File Manager for batch file processing.

The CLI Mode provides a straightforward text-based input mechanism that enables users to directly enter genomic sequence data into a dedicated text area (Supplementary Fig. 9). The interface demonstrates real-time data type detection capabilities, automatically identifying the input format based on both file extensions and content analysis. The system recognizes multiple distinct data types including ’Multi-FASTA’ for multiple sequence files, ’FASTA’ for single sequences, ’FASTQ’ for quality score data, ’PackagedFASTQ’ for compressed quality data, ’DNA’ and ’RNA’ for nucleotide sequences, ’AminoAcids’ for protein sequences, ’NUM’ for numerical data, ’BIN’ for binary information, ’Group’ for grouped data structures, and ’TEXT’ for general text content. The data type indicator updates automatically upon input detection, as demonstrated with the “Multi-FASTA” identification in the example, providing immediate feedback to users about format compatibility with available genomic tools.

**Fig. 4:**
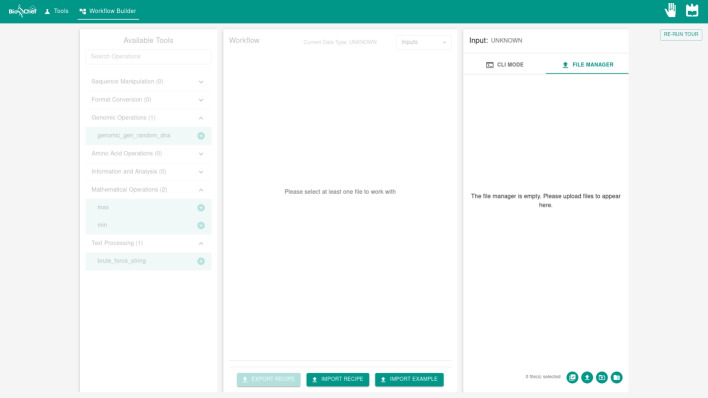
File Manager interface in initial empty state showing upload options and workflow validation message requiring file selection

The File Manager mode implements a file management system designed for batch processing workflows with multiple input files (Fig. 4). In its initial state, the File Manager displays an empty workspace with the message “The file manager is empty. Please upload files to appear here,” while the workflow panel indicates “Please select at least one file to work with.” This design enforces workflow validation by disabling interface actions until appropriate input files are provided, with the exception of the import workflow functionality that enables restoration of previously saved workflow configurations.

The File Manager provides four distinct upload mechanisms through dedicated icons in the interface: individual file upload, batch file selection, folder upload with structure preservation, and ZIP archive extraction. The folder upload functionality maintains the original directory structure, as demonstrated in the Supplementary Fig. 6, where the “test files” folder contains multiple genomic files organized hierarchically. The interface supports file selection through checkboxes, with selected files indicated by green checkmarks and available for workflow processing.

Selected files become available through a dropdown menu in the workflow panel (Supplementary Fig. 10), enabling users to specify which input file should be used for displaying intermediate results during workflow execution. This functionality supports workflow debugging and result validation by allowing users to trace data transformations through the processing pipeline using specific input files.

The File Manager implements additional file and folder management capabilities (Supplementary Fig. 7). Folder operations include creating new files, new folders, and new ZIP archives within the directory structure, as well as deletion capabilities for removing entire folder hierarchies. File-specific operations provide access to content viewing, detailed file information, and individual file deletion. These management features enable users to organize their genomic datasets efficiently within the platform’s file system while maintaining workflow-ready file structures.

The content viewing functionality provides immediate access to file contents through a dedicated viewer interface (Supplementary Fig. 8a), enabling users to examine genomic sequences directly within the platform. The file information system displays metadata including file name, data type, file size, and relative path within the file system hierarchy (Supplementary Fig. 8b).

The File Manager implements validation mechanisms to ensure workflow integrity and prevent processing errors. The system enforces data type consistency across selected files, displaying the notification “Selected files must have the same type” when users attempt to select files with incompatible formats. Similarly, folder-level validation prevents selection of directories containing mixed file types, with notifications such as “Folder contains multiple file types. Please select files with the same type” or “Folder contains files of a different type from the selected files” when conflicts arise. Additionally, the system validates file format compatibility, displaying the notification “Unsupported file FILENAME with type FILETYPE” when users attempt to upload files with unrecognized or incompatible data types. Performance protection mechanisms include file size limitations, with the system loading only the first 10,000 lines of large files and displaying the warning “The file FILENAME is too large. Only the first 10,000 lines will be loaded” to maintain browser stability while preserving usability for substantial genomic datasets.

#### Workflow tool integration

The workflow construction system implements intelligent tool filtering and dynamic workflow management to ensure data type compatibility and prevent processing errors. As demonstrated in the Supplementary Fig. 10, the workflow panel displays the current data type status, which initially corresponds to the input data type when the workflow is empty. This current data type serves as the foundation for the system’s intelligent tool filtering mechanism.

The Available Tools panel implements real-time filtering based on the current workflow data type, displaying only tools that accept the current data type as input (Supplementary Fig. 11). This intelligent filtering mechanism serves as a primary error prevention strategy, ensuring users can only select tools that are compatible with the current workflow state. The example demonstrates how tools in the Sequence Manipulation and Format Conversion categories are presented when the current data type is “Multi-FASTA,” with each tool showing clear input and output format specifications through hover interactions.

Tool integration into the workflow requires only a simple click interaction on the desired tool from the Available Tools panel. Upon selection, the tool is automatically added to the workflow as a dragable component (Supplementary Fig. 12). The workflow immediately executes the added tool with the chosen input data, keeping displaying the current data type as “Multi-FASTA”.

The system demonstrates dynamic responsiveness to workflow changes through real-time tool filtering updates. When a tool that converts data types is added to the workflow, the Available Tools panel immediately updates to reflect the new current data type (Supplementary Fig. 13). The example shows the addition of the fasta_to_seq tool, which converts Multi-FASTA input to DNA format. Upon this conversion, the system displays a notification “Data type updated to DNA” and automatically refreshes the Available Tools panel to show only tools compatible with DNA input, including tools from Format Conversion and Genomic Operations categories. This dynamic filtering ensures continuous workflow validity while providing users with contextually appropriate tool options at each workflow stage.

#### Workflow panel management and execution

The workflow panel serves as the central hub for workflow execution, result visualization, and workflow management operations. When input data is selected and a valid workflow is constructed, the system performs real-time execution, providing immediate feedback and result visualization capabilities.

Upon workflow construction with valid input data, the system immediately executes all workflow steps and displays results through an interactive interface (Supplementary Fig. 14). Each tool component within the workflow displays as a draggable card containing the tool name, control icons, and a “VIEW” button for accessing intermediate results. The intermediate output sections can be expanded or collapsed to facilitate workflow review, showing processed data at each step of the pipeline. The workflow panel concludes with a dedicated “Output” section that displays the final result of the complete workflow execution, providing users with immediate access to the processed genomic data.

The workflow panel supports mid-workflow tool insertion through a hover-based interface design (Supplementary Fig. 15). When users position the cursor between existing tool components, a subtle insertion zone becomes visible, displaying an “Add Tool” button, that when clicked shows above the Available Tools panel the instructional message “Adding a new tool to the workflow. Click on a tool to insert it at the selected position or cancel.” This space-efficient design preserves the clean workflow interface while providing accessible modification capabilities. Upon activation of the insertion mode, the Available Tools panel dynamically filters to display only compatible tools at that specific workflow position. The system evaluates both the output type of the preceding tool and the input requirements of the subsequent tool to ensure workflow validity. Users can exit the insertion mode by clicking the “cancel” button in the notification message above the Available Tools panel, returning to the standard workflow view.

Each workflow tool provides integrated documentation through help icons that display detailed usage information when hovered (Supplementary Fig. 16). The help content is dynamically generated by executing the tool with the -h flag, providing users with authentic command-line documentation including usage syntax, basic options, input/output format specifications, and practical examples. This contextual help system enables users to understand tool functionality without leaving the workflow interface. In addition, each tool may display two auxiliary icons “info” and “error” which appear only when the tool produces informational or error messages during execution. These messages are revealed through hover-based tooltips, providing users with additional and often relevant information about each tool s execution in the case of the “info” icon, and explicit feedback on misconfigurations or incorrect usage through the “error” icon.

The workflow panel implements save options available for both intermediate results and final output. Each tool component includes a save button that downloads the processed result at that specific workflow stage. The download behavior adapts to the input mode: CLI Mode generates single file downloads, while File Manager Mode creates ZIP archives containing multiple processed files when multiple input files are present. This flexible export system supports both individual result preservation and batch processing workflows.

Workflow modification is supported through granular deletion controls accessible via each tool’s menu interface (Supplementary Fig. 17). Users can choose between “Remove this operation” to delete a single tool or “Remove from here downwards” to eliminate the selected tool and all subsequent workflow steps. These deletion options provide precise control over workflow structure while maintaining data flow integrity.

The workflow panel implements validation mechanisms to prevent invalid workflow configurations (Supplementary Fig. 18). When users attempt deletions that would result in incompatible data flow, such as removing the fasta_to_seq tool while maintaining the genomic_complement tool that requires DNA input, the system preserves the current workflow state and displays the error message “Invalid operation: resulting workflow has incompatible steps” (Supplementary Fig. 18a). Similarly, drag-and-drop reordering operations are validated in real-time, with invalid arrangements automatically reverted to the previous valid state while presenting error notifications to inform users of the incompatibility (Supplementary Fig. 18b). This validation system ensures workflow integrity by preventing data type mismatches between consecutive tools.

#### Workflow persistence and reproducibility

The workflow construction interface provides export and import capabilities designed to ensure workflow reproducibility, sharing, and integration with external computational environments. These persistence mechanisms support both complete workflow preservation and partial workflow extraction for modular analysis development.

The export functionality is accessible through two distinct mechanisms that provide flexibility in workflow preservation scope. The primary export option utilizes the “EXPORT RECIPE” button located below the final workflow output, enabling complete workflow preservation including all constructed tools and their configurations. Additionally, the system supports partial workflow export through hidden “Export Previous Steps” buttons positioned between workflow tools, accessible when hovering between tool components. This granular export capability allows users to extract workflow segments up to specific processing stages, facilitating modular workflow development and intermediate result preservation.

Upon activation of either export mechanism, the system presents a export modal (Supplementary Fig. 19) offering three distinct export formats to accommodate different usage scenarios. The “Copy Command” option generates a single-line command pipeline that concatenates all workflow tools using standard UNIX pipe notation, enabling immediate execution in local terminal environments. This format provides the most direct approach for users familiar with command-line genomic analysis workflows and supports immediate local execution without additional setup requirements.

The script generation option creates bash scripts that include workflow execution logic, dependency management, and safety checks. These generated scripts automatically handle GTO toolkit installation, provide interactive user prompts for workflow confirmation, support both single-file and batch processing modes depending on the input configuration, and include error handling and temporary file management. The scripts adapt dynamically to the input mode, generating single-file processing logic for CLI Mode workflows or sophisticated batch processing capabilities for File Manager Mode workflows with multiple input files.

The “Copy Command” and script generation export options collectively provide an essential solution to the browser-based computational limitations referenced in the file management system, where large files are restricted to the first 10,000 lines to maintain browser stability. These export formats ensure that users can leverage BioChef’s workflow construction capabilities for prototyping and validation with sample data, then seamlessly transition to local execution environments for processing complete large-scale genomic datasets without size restrictions.

The configuration file export generates structured JSON documents that preserve complete workflow metadata including tool sequences, parameter configurations, input data specifications, and creation timestamps. These configuration files enable precise workflow reproduction and facilitate workflow sharing between users while maintaining complete fidelity to the original workflow construction.

The import functionality, accessible through the “IMPORT RECIPE” button positioned below the workflow output section, provides workflow restoration capabilities through a dedicated import modal (Supplementary Fig. 20). The import system supports two primary restoration methods: command line parsing and configuration file restoration. The command line import option enables users to input workflow commands using standard UNIX pipe notation, with the system automatically parsing tool sequences, parameter configurations, and data flow relationships to reconstruct the corresponding workflow structure. The configuration file import provides precise workflow restoration from previously exported JSON files, ensuring complete fidelity in workflow reproduction including parameter values, tool ordering, and metadata preservation.

These persistence mechanisms collectively ensure workflow reproducibility across different computational environments while supporting collaborative genomic analysis development through standardized export formats compatible with existing bioinformatics infrastructure. The combination of immediate execution commands, scripts, and structured configuration files addresses diverse user requirements ranging from quick local testing to formal workflow documentation and sharing.

### Browser compatibility assessment

To ensure broad accessibility across different computing environments, BioChef underwent browser compatibility testing using Playwright [37] testing framework. The testing process involved manual evaluation of the platform’s functionality across major browser engines including Chromium, WebKit, and Firefox, representing the dominant browser technologies used globally.

On the Tools Page, every available tool was selected and executed one-by-one using valid inputs, verifying that execution completed and produced the expected output. On the Workflow Page, a predefined recipe containing four different tools was first imported, using CLI input mode, and confirmed that the workflow could be imported and executed end-to-end. Workflow editing operations were then tested, such as inserting a tool at the end and in the middle of the workflow, deleting tools, and the drag-and-drop functionality, verifying that the workflow updated correctly and could still be executed. To assess batch processing behavior, the input mode was switched to File Explorer and the input ingestion was tested via ZIP archives, folders, and individual files, selecting multiple files for execution. Finally, all supported export options were verified that functioned correctly by exporting the workflow through each available modality and confirming that the expected export artifacts were generated and could be downloaded and/or re-imported where applicable.

Results demonstrate excellent compatibility across all tested browser engines. Chromium and Firefox exhibited complete functionality with all features operating as designed. WebKit testing on Windows 10 demonstrated full compatibility with core workflow construction and tool execution features, with file upload and download functionality being untestable due to WebKit-Windows integration limitations. Additional testing using GNOME Web, which utilizes the WebKit engine on Linux, confirmed complete functionality including file operations, validating full WebKit compatibility across platforms.

The successful cross-browser compatibility validates BioChef’s design approach using standards-compliant web technologies. Since BioChef functions effectively across Chromium, WebKit, and Firefox engines, the platform is compatible with all major modern browsers including Chrome, Firefox, Safari, Opera, and Edge (Table 1). The platform’s reliance on widely-supported technologies including ReactJS, WebAssembly, and standard web APIs ensures consistent functionality across these browser ecosystems used in academic and clinical research environments. This broad compatibility eliminates platform-specific installation requirements and enables immediate access to genomic analysis capabilities regardless of users’ preferred browser or operating system.

**Table 1:** BioChef browser compatibility assessment results

Browser	Engine	Compatibility
Chrome	Chromium	$\checkmark$
Firefox	Gecko	$\checkmark$
Safari	WebKit	$\checkmark$
Edge	Chromium	$\checkmark$
Opera	Chromium	$\checkmark$
GNOME Web	WebKit	$\checkmark$

### Application initialization performance

To evaluate the user experience during application startup, were conducted systematic measurements of Web Vitals metrics using Chrome DevTools Performance profiler. Ten independent sessions were recorded, each involving a page refresh followed by a single user interaction to assess initial responsiveness.

The application demonstrates consistent initialization performance across multiple sessions (Table 2). The Largest Contentful Paint (LCP) averaged 0.583 0.011 s, indicating rapid visual content delivery that meets Google’s “Good” performance threshold. The Interaction to Next Paint (INP) averaged 30.5 4.2 milliseconds, demonstrating highly responsive initial user interactions. Cumulative Layout Shift (CLS) remained consistently minimal at 0.01, indicating excellent visual stability during application loading.

Total initialization time averaged 2.31 0.35 s, with some variability attributable to browser caching behavior. These measurements reflect the time required for the React application and user interface components to load and become interactive, prior to any WebAssembly module loading which occurs on first tool usage.

The results demonstrate that BioChef achieves excellent application bootstrap performance, ensuring users can quickly access the interface and begin workflow construction without delay.

**Table 2:** Application initialization performance metrics (n=10)

Metric	Mean	Std Dev	Range	Google threshold
LCP (s)	0.583	0.011	0.572–0.60	$< 2.5$ (Good)
INP (ms)	30.5	4.2	22–35	$< 200$ (Good)
CLS	0.01	0.00	0.01	$< 0.1$ (Good)
Total (s)	2.31	0.35	1.662–2.95	2–

### WebAssembly module loading performance

To assess the user experience during first-time tool usage, we measured the loading times for individual WebAssembly modules across the GTO toolkit. These measurements capture the overhead associated with dynamic loading of pre-built static assets, WebAssembly bytecode instantiation, and runtime initialization that occurs when users first select a genomic tool for analysis.

Loading time measurements were conducted using the browser’s Performance API integrated into the module loading mechanism. A total of 62 individual tool loading events were recorded during normal application usage, representing the complete range of available genomic analysis tools. Each measurement encompasses the complete loading process: fetching pre-generated wrapper scripts, loading pre-compiled WebAssembly modules containing embedded bytecode, just-in-time compilation of bytecode to native machine code for the target CPU architecture, Emscripten runtime environment initialization, and making tool functions available to the JavaScript runtime.

The WebAssembly module loading performance demonstrates consistent characteristics across the toolkit (Table 3). Loading times averaged 181.5 36.2 milliseconds, with individual measurements ranging from 131 to 291 milliseconds. The median loading time of 172 milliseconds indicates that most tools load efficiently, while the interquartile range of 152–209 milliseconds demonstrates relatively low variability in loading performance across different tools.

These loading times represent a one-time overhead per tool, as subsequent usage of the same tool benefits from browser caching and module reuse. The sub-200-millisecond average loading time ensures that users experience minimal delay when first accessing genomic analysis capabilities, contributing to the platform’s overall responsiveness and usability.

**Table 3:** WebAssembly module loading performance (n=62)

Metric	Value (ms)
Mean	181.5
Standard deviation	36.2
Median	172.0
Min-max range	131–291
Interquartile range	152–209

### Local vs. platform performance evaluation

To quantitatively assess the computational overhead introduced by WebAssembly execution compared to native binary performance, we conducted systematic performance measurements of identical genomic workflows executed both locally and within the BioChef platform. This evaluation provides empirical evidence of the platform’s practical viability for real-world genomic analysis tasks.

Performance measurements were conducted on a system equipped with an AMD Ryzen 7 4800 H processor (8 cores, 16 threads) with Radeon Graphics and 16.0 GB of memory, running Ubuntu 24.04.2 LTS.

The test workflow consisted of a six-step genomic analysis pipeline: complement calculation, mutation simulation with 50% error rate, FASTA to raw sequence conversion, extraction of the first 1000 nucleotides, sequence format reconstruction, and sequence reversal.

Four genomic datasets were obtained from NCBI Datasets  [38] representing diverse organisms: *Alligator mississippiensis*, *Branchiostoma lanceolatum*, *Hydrolagus colliei*, and *Homo sapiens*. To ensure fair comparison given browser memory constraints, both local and platform tests utilized identical datasets limited to the first 10,000 lines of each genome file, with all files standardized to 0.81 MB after truncation. Typical datasets range from a few thousand to several million lines. In our testing, the platform was able to handle larger inputs but the 10,000 lines limit was selected here as a conservative value that allows testing workflows while remaining practical to run inside the browser.

For local execution measurements, the workflow pipeline was executed directly using the compiled C binary tools, with GNU time capturing runtime and memory usage metrics. Platform measurements employed Selenium WebDriver [39] to automate the BioChef web interface, simulating realistic user interactions. The testing script monitored Chrome process memory throughout workflow execution, establishing a baseline memory footprint before workflow initiation and tracking memory growth during processing. This approach ensured fair comparison by measuring only the memory overhead attributable to workflow execution rather than the entire browser footprint. The automated test sequence included navigating to the workflow page, importing the pre-configured workflow JSON, uploading test data, and monitoring execution until completion. Both local and platform test configurations were executed three times to calculate mean values.

**Fig. 5:**
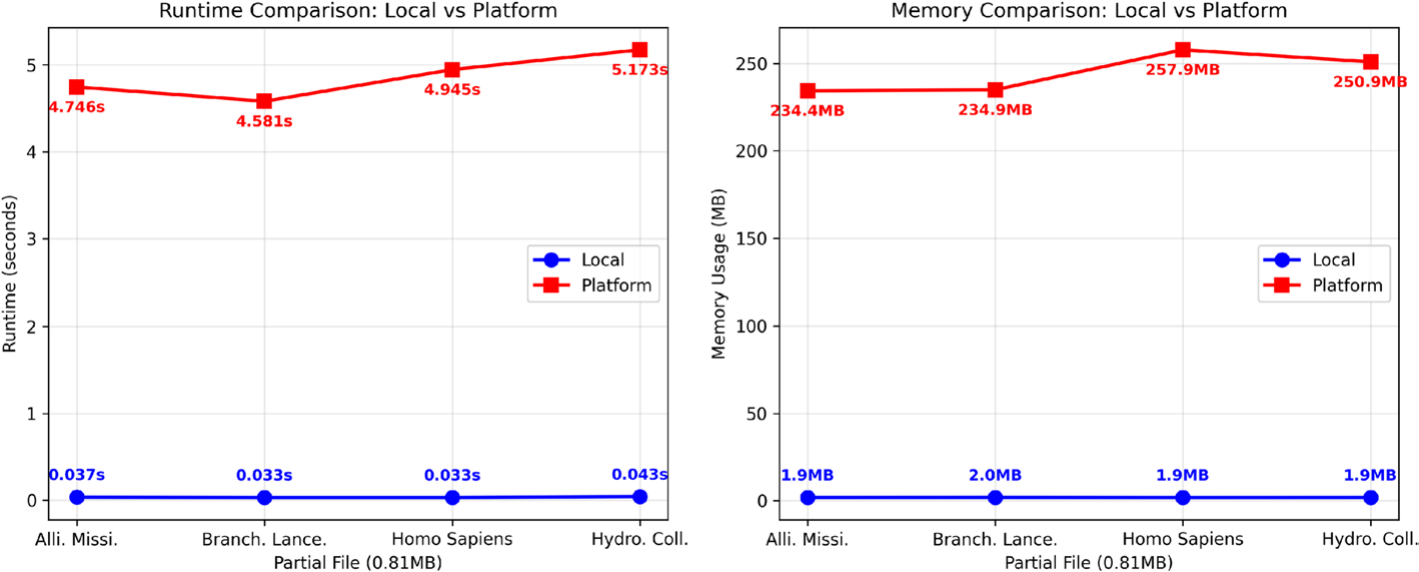
Performance comparison between local and platform execution of the genomic workflow. (Left) Runtime comparison showing exponential scaling for platform execution versus near-constant local performance. (Right) Memory usage comparison demonstrating linear scaling for platform execution with higher baseline due to browser overhead

The performance evaluation revealed distinct characteristics between local and platform execution, as illustrated in Fig. 5. Runtime performance demonstrated significant overhead for platform implementation, with execution times ranging from 4.58 to 5.17 s compared to local execution times of 0.033 to 0.043 s. This represents approximately 130 slower execution for platform implementation, reflecting the overhead inherent in WebAssembly execution within browser environments. Note that local execution times exclude any installation or setup time which for a first-time user could make the platform faster overall.

Memory consumption patterns exhibited different scaling characteristics. Local execution maintained consistent memory usage around 1.9 MB across all dataset sizes, while platform memory consumption showed moderate variation from 234.4 MB to 257.9 MB.

The performance overhead can be attributed to several factors inherent in WebAssembly execution within browser environments. JavaScript-to-WebAssembly boundary crossings introduce latency for each tool invocation in the pipeline. The browser’s sandboxed execution model imposes additional security checks and memory management overhead. File system emulation through Emscripten’s virtual file system adds translation costs for I/O operations. The evaluation script measures the memory usage right before execution of the workflow and compares the final memory value with this baseline measurement to try to get an reading as accurate as possible. It is also important to consider that memory measurements on the platform may include usage from other browser-related processes beyond the workflow itself. Although the evaluation script accounts for baseline memory before execution, the memory growth is measured at the process level and may capture additional browser activities not directly related to the workflow (e.g., rendering tasks, garbage collection, or Chrome internal services). This introduces a potential source of noise, meaning that the reported platform memory usage might slightly overestimate the true memory footprint of the workflow execution alone.

### Performance comparison with galaxy workflow management system

To evaluate BioChef’s performance characteristics against established bioinformatics platforms, was conducted a comparative analysis with Galaxy [13], selected as the primary comparison platform due to its similar workflow construction capabilities and widespread adoption in genomics research. The comparison utilized an identical four-step workflow (FASTA Complement $\rightarrow$ FASTA Reverse $\rightarrow$ FASTA Split $\rightarrow$ FASTA Extract) executed on the same genomic dataset (partial *Branchiostoma lanceolatum* genome) to ensure fair performance assessment. Galaxy was benchmarked on a shared/public deployment; runtimes therefore represent wall-clock time under typical multi-user conditions, including any job submission and queueing delays.

**Fig. 6:**
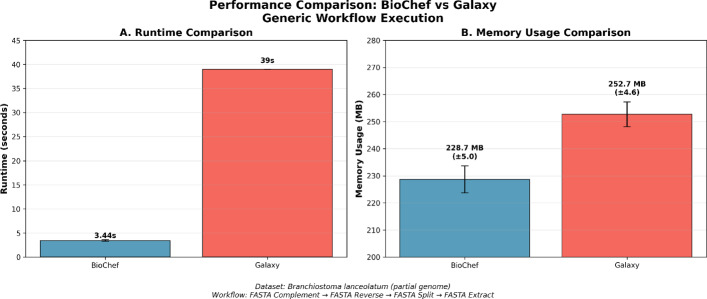
Performance comparison between BioChef and Galaxy for genomic workflow execution. (A) Runtime comparison showing BioChef’s 11.3 faster execution for the four-step workflow. (B) Memory usage comparison demonstrating similar resource requirements between platforms

The performance comparison, shown in Fig. 6, reveals distinct execution characteristics between client-side WebAssembly execution and server-based processing. BioChef’s integrated workflow execution completed in $3.44 \pm 0.17$ seconds, demonstrating consistent performance across multiple runs with minimal variance (coefficient of variation: 5.0%). In contrast, Galaxy’s server-based execution required a cumulative runtime of 39 s (7 + 13 + 12 + 7 s for individual jobs), representing an 11.3$\times$ longer execution time compared to BioChef’s browser-based implementation.

The observed difference in performance can be attributed to several architectural factors inherent in server-based workflow systems. Galaxy’s execution model includes job queuing, resource allocation, inter-job communication overhead, and file system I/O operations between discrete workflow steps. Each Galaxy job requires individual startup and teardown phases, container initialization, and intermediate file writing/reading operations that contribute substantially to the overall workflow execution time.

Memory consumption analysis reveals comparable resource requirements between the two platforms, with distinct allocation strategies. BioChef’s browser-based execution utilized $228.7 \pm 5.0$ MB of memory. Galaxy’s individual jobs consumed between 246.4-−257.4 MB of maximum memory per step, with peak memory usage of 257.4 MB during the FASTA Split operation.

The performance comparison highlights fundamental differences in workflow execution paradigms. Galaxy’s job-based architecture provides advantages for large-scale computational workflows through resource isolation, fault tolerance, and distributed execution capabilities. However, the overhead associated with job management, queuing systems, and inter-step communication creates substantial latency penalties.

BioChef’s integrated client-side execution eliminates these overhead sources by maintaining workflow state in memory and executing tools within a continuous WebAssembly runtime environment. This approach trades the scalability advantages of server-based systems for improved responsiveness in interactive analysis scenarios, making it particularly suitable for workflow prototyping, educational applications, and moderate-scale research tasks where immediate feedback is prioritized over maximum computational throughput.

## Discussion

Table 4 provides an overview of platform distinctions in bioinformatics workflow management, highlighting how BioChef uniquely combines client-side execution, WebAssembly technology, custom workflow construction for genomic manipulation and analysis, and installation-free access for users, a combination not achieved by any existing related work in the bioinformatics domain.

**Table 4:** Comparison of BioChef with related platforms and tools

Platform	Client-side	WASM	Custom workflow	No install
**BioChef**	$\checkmark$	$\checkmark$	$\checkmark$	$\checkmark$
ViralWasm [16]	$\checkmark$	$\checkmark$	✗	$\checkmark$
kana [17]	$\checkmark$	$\checkmark$	✗	$\checkmark$
UBiT2 [30]	$\checkmark$	✗	✗	$\checkmark$
Chromatic [27]	$\checkmark$	$\checkmark$	✗	$\checkmark$
fastq.bio [29]	$\checkmark$	$\checkmark$	✗	$\checkmark$
sandbox.bio [28]	$\checkmark$	$\checkmark$	✗	$\checkmark$
Galaxy [13]	✗	✗	$\checkmark$	$\checkmark$
BioWMS [33]	✗	✗	$\checkmark$	$\checkmark$
BioDepot [31]	✗	✗	$\checkmark$	✗
Playbook [32]	✗	✗	$\checkmark$	$\checkmark$
Closha 2.0 [34]	✗	✗	$\checkmark$	$\checkmark$

BioChef represents the first client-side workflow builder specifically designed for genomic data manipulation and analysis using WebAssembly technology. The platform compiles the GTO toolkit to WebAssembly and provides a drag-and-drop interface designed to lower the barrier to using command-line genomic tools, while ensuring complete data privacy through local processing.

The platform addresses several critical limitations observed in current bioinformatics workflow systems through its unique architecture. Existing WebAssembly-based platforms like ViralWasm, kana, and UBiT2 restrict users to predefined workflows with limited customization capabilities. While these tools demonstrate the technical feasibility of browser-based genomic analysis, they cannot accommodate the multi-step workflows that characterize modern genomic research, nor do they support genomic manipulation operations that are fundamental to DNA analysis workflows. BioChef transcends these limitations through its drag-and-drop interface that enables arbitrary workflow construction with complete flexibility for diverse genomic analysis requirements.

Traditional server-based solutions introduce fundamental privacy and infrastructure challenges. Galaxy, BioWMS, and Closha 2.0 require data upload to external servers, creating vulnerabilities during transmission and storage while raising compliance issues with regulatory frameworks such as HIPAA and GDPR. These platforms necessitate substantial infrastructure requirements and technical expertise for deployment and maintenance. BioChef eliminates these concerns through client-side execution that keeps sensitive genomic data local while requiring no specialized setup or infrastructure investment.

The installation-free nature of BioChef contrasts sharply with platforms like BioDepot that require substantial user setup, including Docker installation, containerization knowledge, and system administration expertise. While BioDepot provides powerful workflow construction capabilities, its setup complexity creates barriers for researchers who lack technical infrastructure or IT support. BioChef eliminates these prerequisites by operating entirely within standard web browsers without requiring any local software installation or configuration.

The platform s implementation using modern web technologies including ReactJS and MaterialUI delivers a clean interface designed for ease of use, that contrasts with the often complex and dated user experiences of traditional bioinformatics platforms. Unlike server-based workflow systems such as Galaxy that execute workflows in batch mode after construction, BioChef provides real-time execution as users build their workflows. This immediate feedback allows researchers to inspect intermediate results at each step, enabling iterative refinement and validation during workflow development. The platform’s error prevention system further enhances usability through real-time data type checking and contextual tool filtering, ensuring workflow validity while preventing incompatible tool combinations before execution. Unlike sequential pipelines, BioChef s interactive mode dynamically restricts tool combinations based on Input/Output compatibility to prevent errors before execution, saving time by avoiding manual iterative debugging.

Cross-browser compatibility testing confirms reliable operation across all major browser engines (Chromium, WebKit, Firefox), ensuring broad accessibility. Application initialization achieves excellent Web Vitals scores (LCP 0.583s, INP 30.5ms), while WebAssembly module loading averages just 181.5ms across 62 tools. Performance comparison with Galaxy reveals BioChef’s integrated client-side execution completing workflows 11.3 faster than server-based processing, highlighting the efficiency advantages of eliminating job queuing and inter-step communication overhead.

Despite these advantages, BioChef faces computational limitations inherent to browser-based execution. The platform demonstrates approximately 130 slower performance compared to native binaries due to WebAssembly sandbox overhead and JavaScript-WebAssembly boundary crossings. Memory consumption patterns also reflect browser execution constraints, with workflows requiring 234–258 MB compared to 1.9 MB for native execution. Additionally, browser environments impose restrictions on data volume processing, as demonstrated in our Galaxy comparison where datasets were limited to ensure browser stability, highlighting the platform’s unsuitability for large-scale genomic analyses that require processing complete genome files.

Although BioChef s memory limitations are a result of inherent browser constraints, they come with the advantage of eliminating the ecological and privacy costs associated with server-side storage. With BioChef s client-side execution, no residual data is stored on external servers, offering an environmental benefit by avoiding unnecessary data duplication, transmission, or indefinite archiving. Data is managed solely by the user, which ensures complete control over privacy. Additionally, this approach eliminates the need for server-side authentication, authorization, or data confidentiality management, further enhancing data security and privacy.

A further limitation of the current BioChef implementation is that the usage is restricted to tools from the GTO toolkit. This constraint reduces flexibility compared to extensible platforms such as Galaxy that support large collections of community-contributed tools.

Future development should focus on addressing these computational limitations through hybrid client–server architecture where lightweight operations remain client-side while intensive analyses are offloaded to high-performance computing resources. Extensibility through plugin-based architecture would enable community-driven integration of domain-specific tools through standardized WebAssembly packages. This includes expanding support beyond the GTO toolkit by integrating additional widely used genomic tools or by providing developer-oriented documentation that demonstrates how new tools can be ported to WebAssembly and incorporated into BioChef. Enhanced visualization capabilities providing immediate graphical feedback on results would improve interpretability and research utility. Advanced workflow logic incorporating conditional branching and decision-based workflows would expand analytical flexibility without compromising usability. Usability testing with biologists and healthcare professionals would provide quantitative metrics to guide interface refinements and ensure the platform effectively balances accessibility with analytical rigor.

Future versions of BioChef could also enable easy export of workflows to established pipeline systems such as Snakemake or Nextflow to enable execution on high-performance computing infrastructures that support them.

These enhancements would advance BioChef from proof-of-concept to production-ready platform, combining the privacy advantages of client-side execution with the scalability requirements of modern genomic research.

## Conclusion

BioChef represents the first client-side visual workflow builder tailored specifically for genomics, demonstrating that WebAssembly execution can successfully address key barriers in genomic data analysis. By compiling the GTO toolkit to WebAssembly and providing a drag-and-drop interface designed to be intuitive, the platform eliminates installation requirements, ensures complete data privacy through local processing, and makes complex bioinformatics operations accessible to non-technical users.

While browser-based execution introduces computational constraints for large-scale datasets, BioChef achieves acceptable performance for moderate-sized genomic data, validating the client-side approach for genomic workflow construction and execution.

This work represents the beginning of a promising research direction that, when expanded with complementary approaches discussed previously, has the potential to become a highly valuable tool for genomic manipulation and analysis. This foundational work establishes the feasibility of browser-based genomic analysis and demonstrates how modern web technologies can bridge the accessibility gap in bioinformatics without compromising data privacy or scientific reproducibility.

## Additional file

Supplementary file 1 (pdf 1054 KB)

## Data Availability

The software (source code, prebuilt WebAssembly binaries, and reproducibility artifacts) is openly available at https://github.com/ieeta-pt/Biochef. An online demo is available at https://ieeta-pt.github.io/Biochef/. Benchmark inputs used in browser-based tests are truncated FASTA excerpts derived from the following genome assemblies deposited in NCBI Datasets (https://www.ncbi.nlm.nih.gov/datasets/): Homo sapiens (GRCh38.p14), RefSeq assembly GCF_000001405.40 (https://www.ncbi.nlm.nih.gov/datasets/genome/GCF_000001405.40/); Alligator mississippiensis, RefSeq assembly GCF_030867095.1 (https://www.ncbi.nlm.nih.gov/datasets/genome/GCF_030867095.1/); Hydrolagus colliei, GenBank assembly GCA_035084275.1 (https://www.ncbi.nlm.nih.gov/datasets/genome/GCA_035084275.1/); Branchiostoma lanceolatum, RefSeq assembly GCF_035083965.1 (https://www.ncbi.nlm.nih.gov/datasets/genome/GCF_035083965.1/). The exact partial FASTA files and JSON workflow snapshots used for benchmarking are provided in the repository under tests/local_test/ and tests/platform_test/, respectively. No new data were generated; all datasets are publicly available.

## Code availability

Online demo: https://ieeta-pt.github.io/Biochef/. Source code, WebAssembly binaries, and reproducibility artifacts: https://github.com/ieeta-pt/Biochef.

## Material availability

Project name: BioChef. Project home page: https://github.com/ieeta-pt/Biochef. Demo: https://ieeta-pt.github.io/Biochef/. Operating system(s): Platform independent (browser-based; tested on Chromium, Gecko, and WebKit engines). Programming language: − C (GTO tools compiled to WebAssembly). − JavaScript/TypeScript (React UI). − Python (build utilities). Other requirements: None to run in the browser beyond a modernWebAssemblyenabled browser. To build from source: Node.js $\ge$ 16, Emscripten $\ge$3.1.65, Python 3.x, and a C toolchain (see repository). License: MIT. Any restrictions to use by non-academics: None.
