# Supplementary material for: Biochef: a client-side WebAssembly-based workflow builder for genomic data analysis
Source: BMC Bioinformatics. 2026 Apr 9;27:103. doi: 10.1186/s12859-026-06431-1 (PMC13173965; doi:10.1186/s12859-026-06431-1)
Supplement: Supplementary file 1 — (pdf 1054 KB) [file 12859_2026_6431_MOESM1_ESM.pdf]

Supplementary Material

Tool configuration and testing

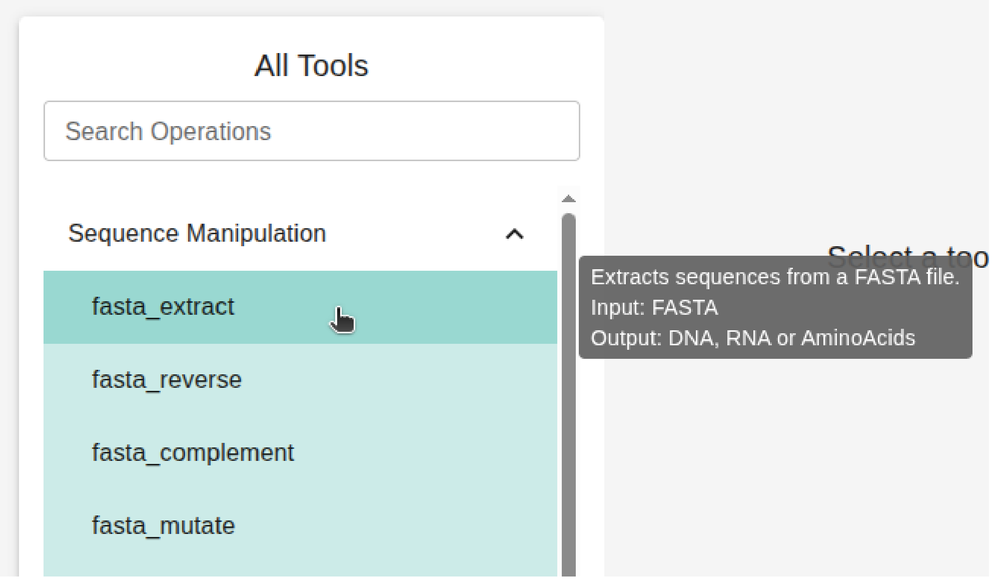

Figure 1: Tool selection with contextual help display for the fasta_extract tool showing input/output format specifications.

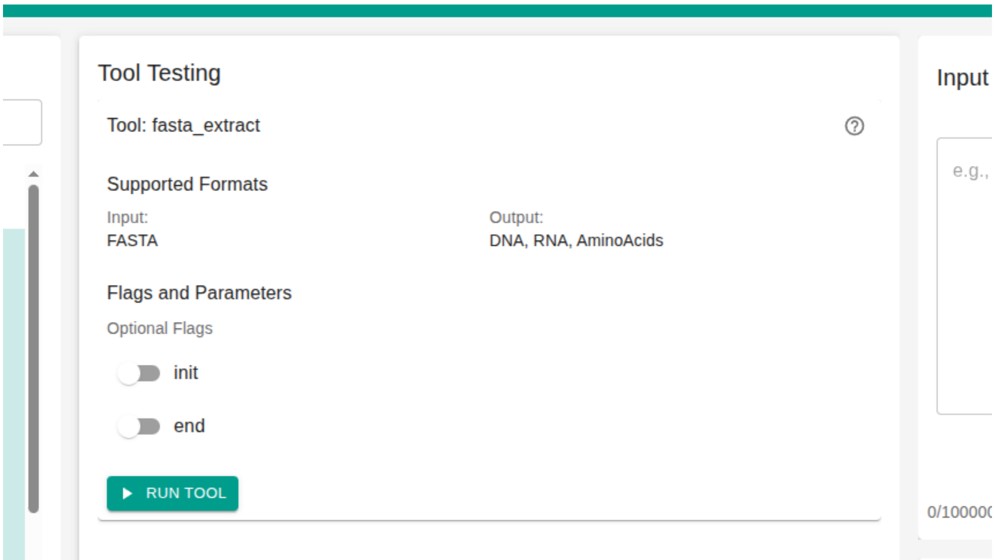

Figure 2: Tool testing panel showing parameter configuration options and input format selection for the selected fasta_extract tool.

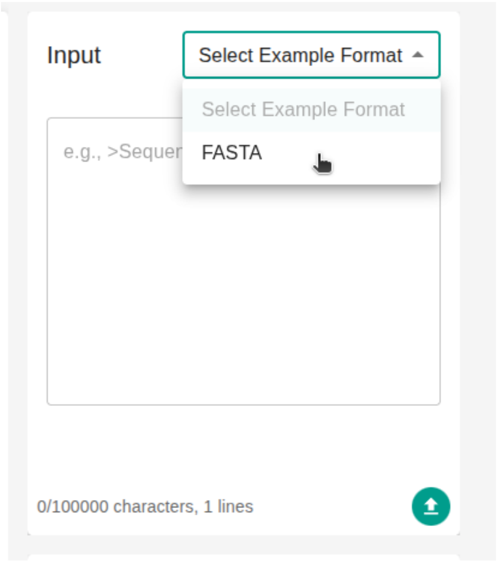

Figure 3: Input sample example selection showing the dropdown menu with FASTA format option for immediate testing.

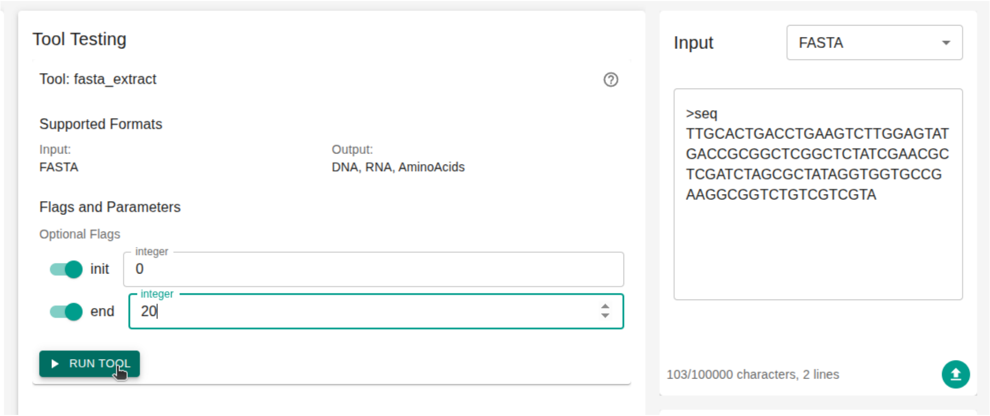

Figure 4: Parameter configuration with example input data loaded and tool execution readiness indicated by the "RUN TOOL" button.

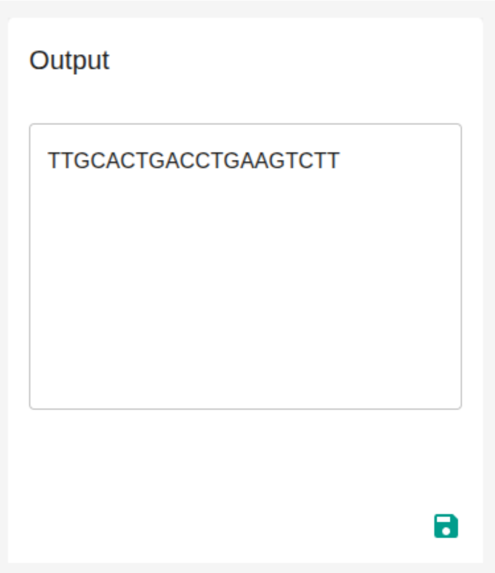

Figure 5: Output display showing processed results with the extracted sequence and integrated download capabilities.

File management and inspection

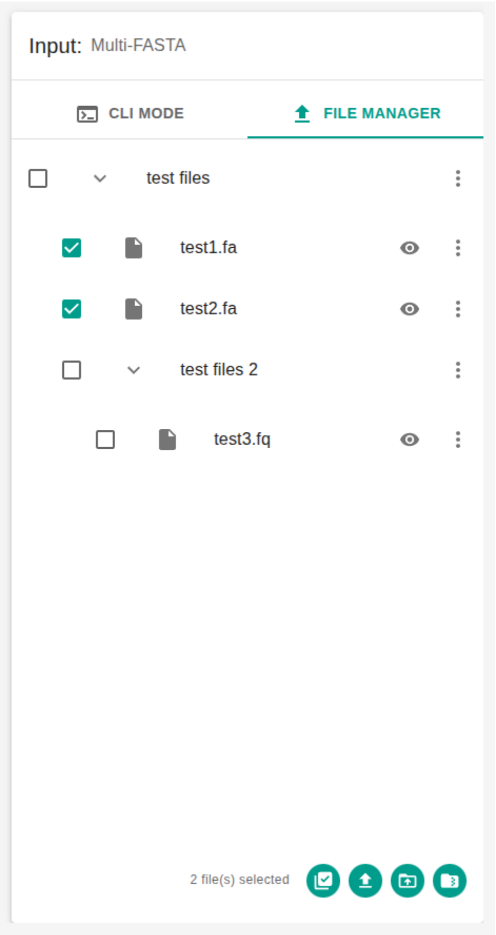

Figure 6: File Manager displaying hierarchical folder structure with selected files of compatible data types ready for workflow processing.

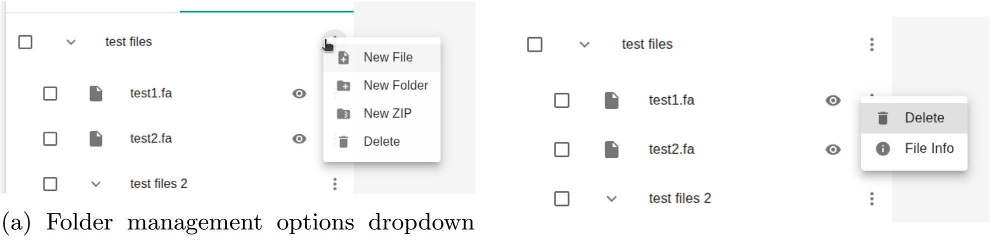

(a) Folder management options dropdown menu

(b) File operation options dropdown menu

Figure 7: File Manager dropdown menus for folder and file operations, accessed through the menu button interface.

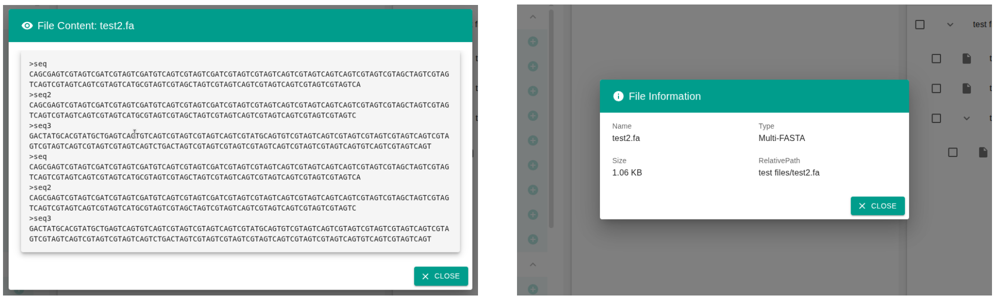

(a) File content viewer displaying Multi-FASTA sequences

(b) File information dialog showing metadata and properties

Figure 8: File Manager inspection capabilities for content review and metadata analysis.

Workflow construction and execution

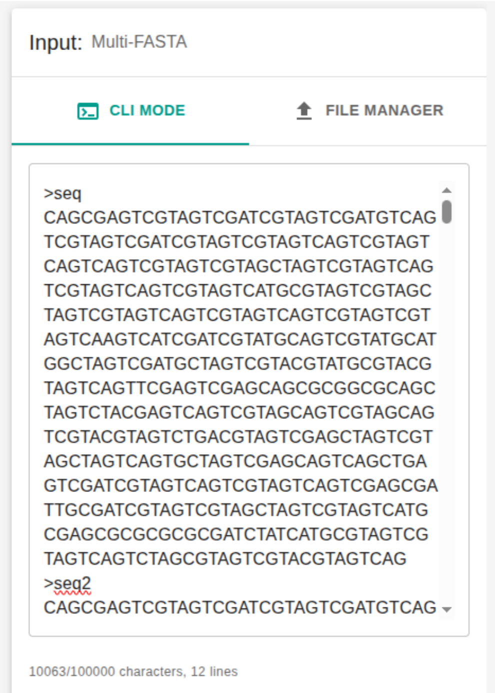

Figure 9: CLI Mode interface showing direct text input with Multi-FASTA example data and automatic data type detection.

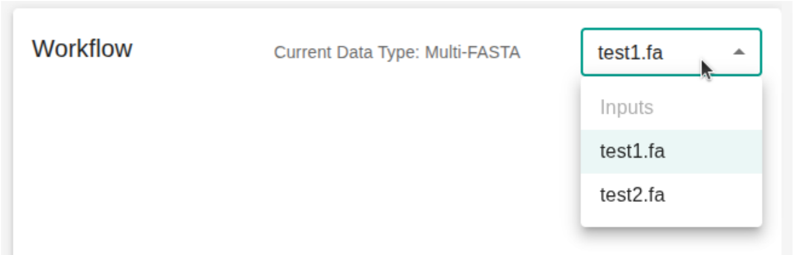

Figure 10: Workflow panel showing input file selection dropdown for specifying which file to use for intermediate result visualization during workflow execution.

Validation behaviors

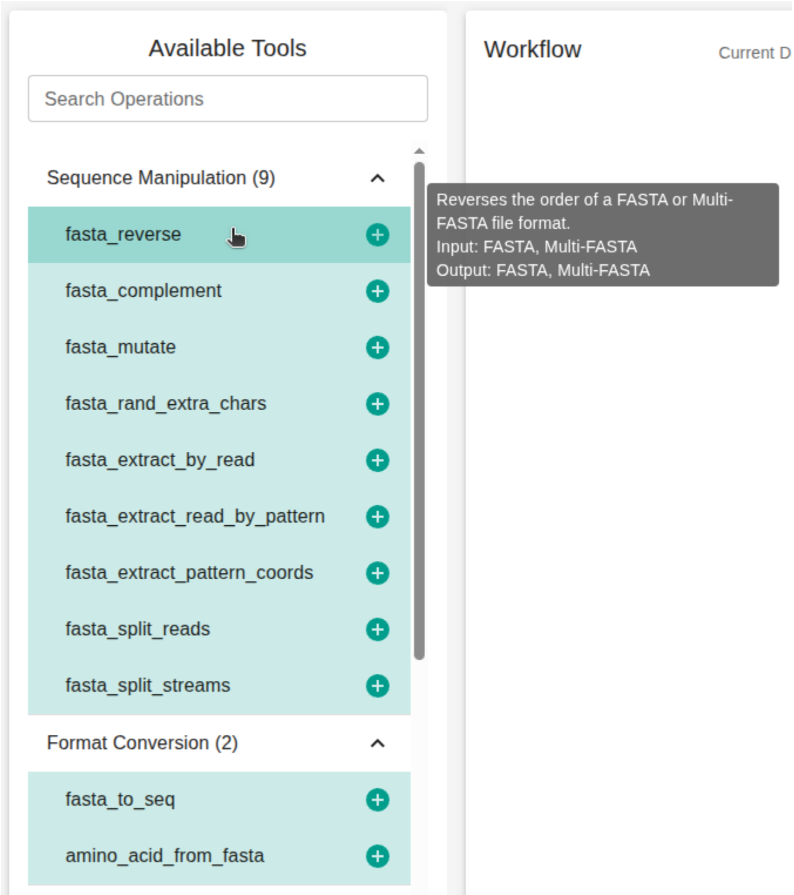

Figure 11: Available Tools panel showing intelligent filtering where only tools compatible with the current data type (Multi-FASTA) are displayed, with hover information for the fasta_reverse tool.

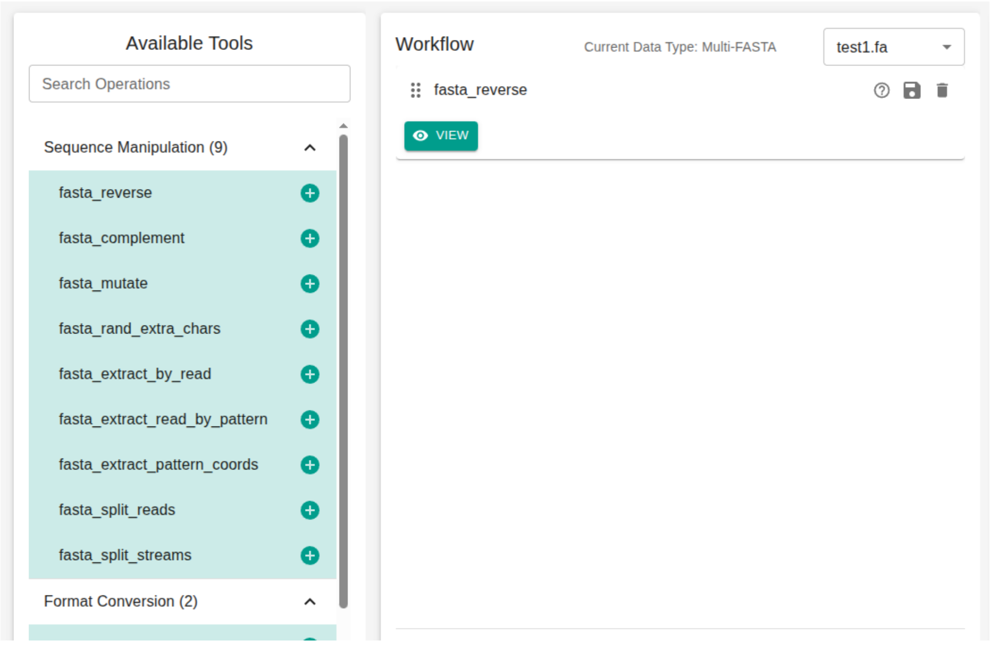

Figure 12: Workflow panel after adding the fasta_reverse tool, showing the tool as a draggable component.

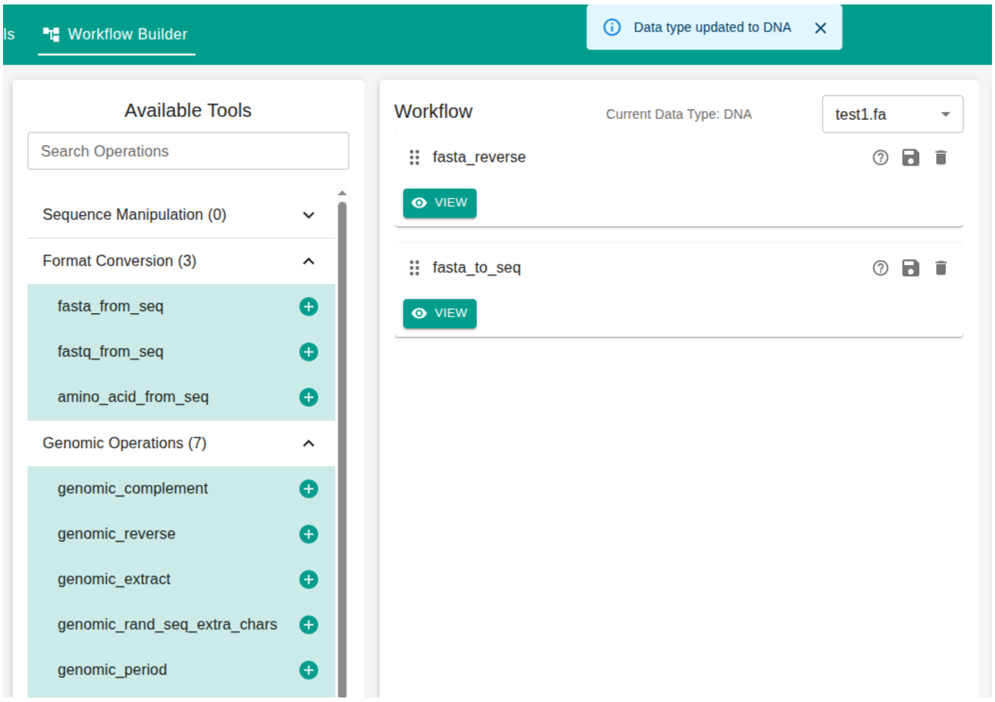

Figure 13: Dynamic tool filtering demonstration showing how Available Tools updates when the workflow data type changes to DNA after adding fasta_to_seq, with a notification indicating the data type change.

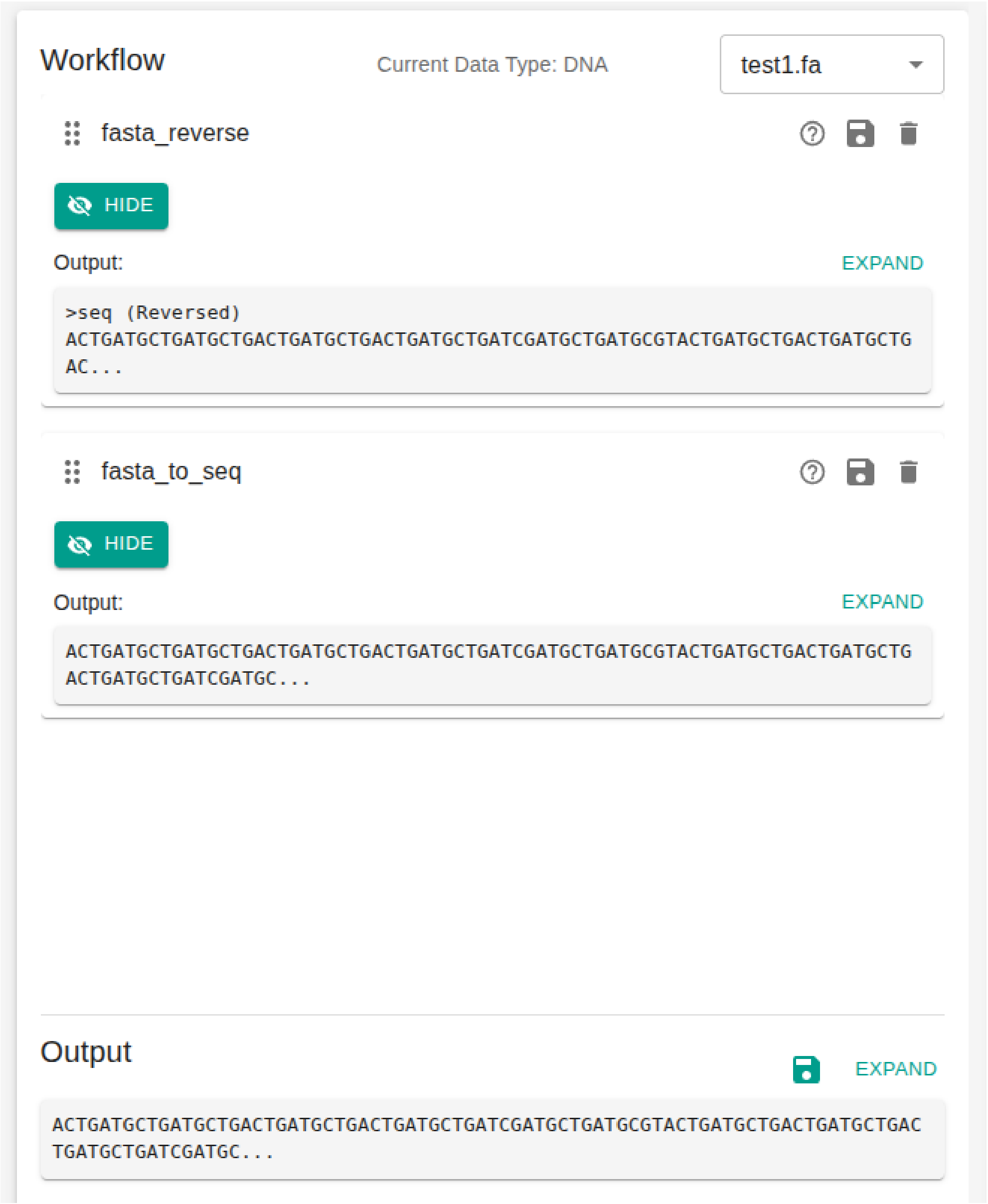

Figure 14: Workflow panel showing real-time execution with intermediate results accessible through VIEW buttons and final output display at the bottom.

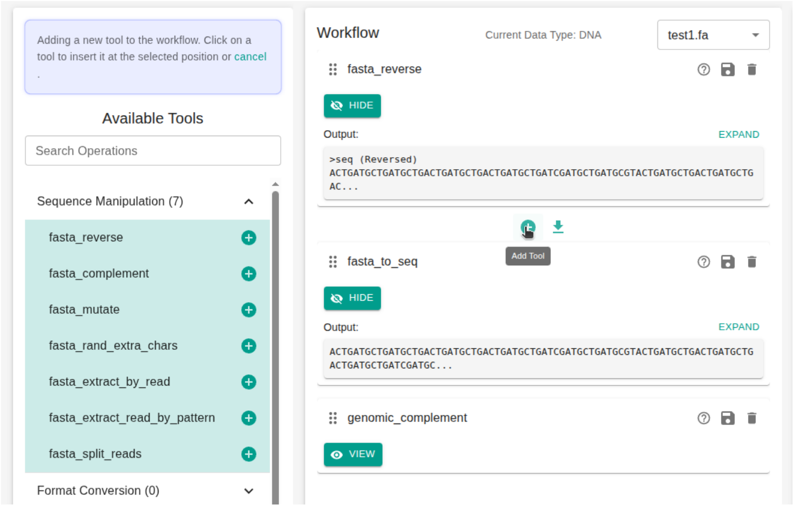

Figure 15: Mid-workflow tool insertion interface showing the activation area between tools and contextual tool filtering in the Available Tools panel.

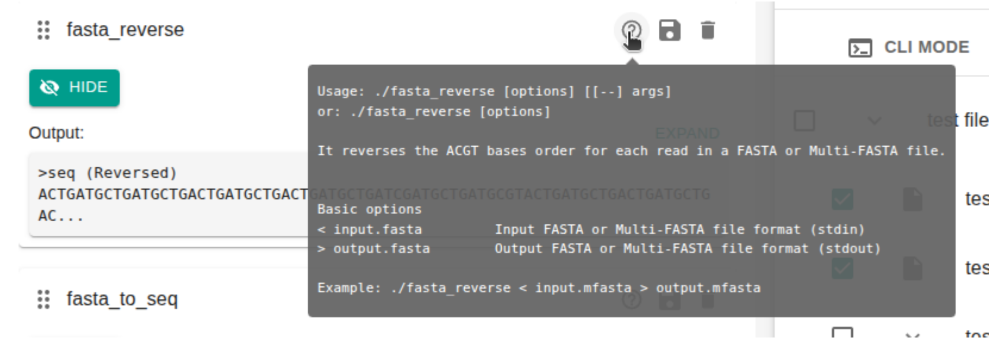

Figure 16: Tool help functionality showing tooltip with usage information accessed through the help icon.

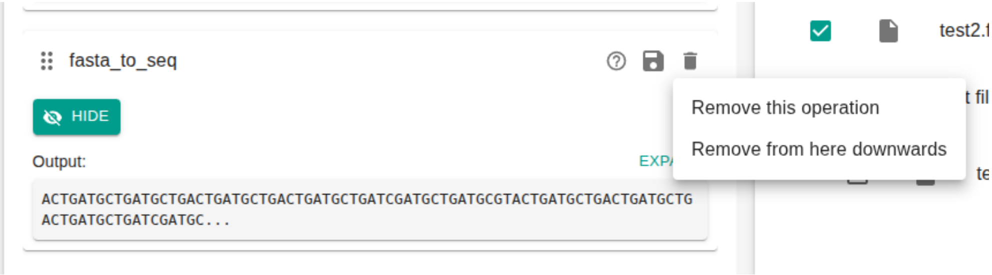

Figure 17: Tool deletion options showing contextual menu with "Remove this operation" and "Remove from here downwards" choices.

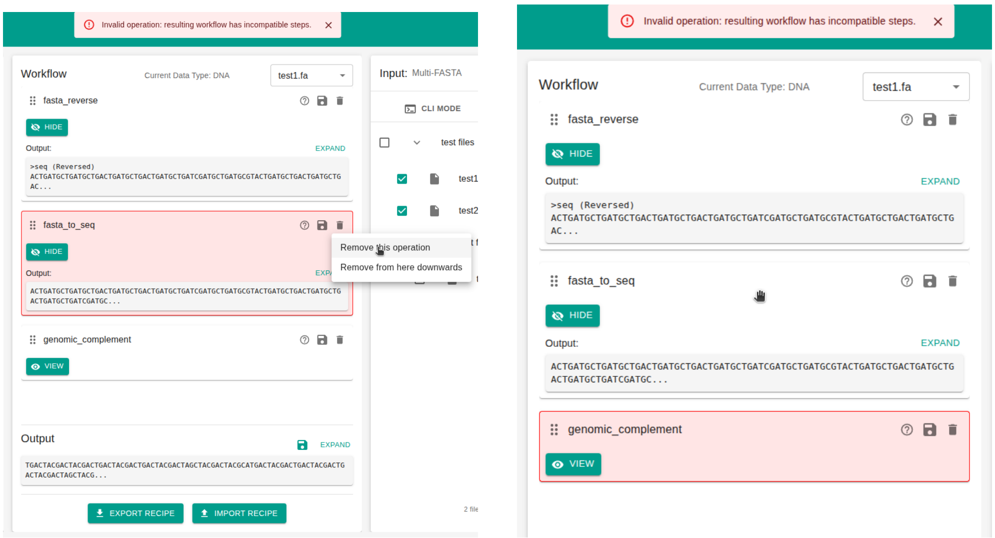

(a) Invalid deletion attempt with error notification

(b) Invalid reordering attempt with automatic reversal

Figure 18: Workflow validation system preventing invalid operations through error notifications and automatic state restoration.

Export and import

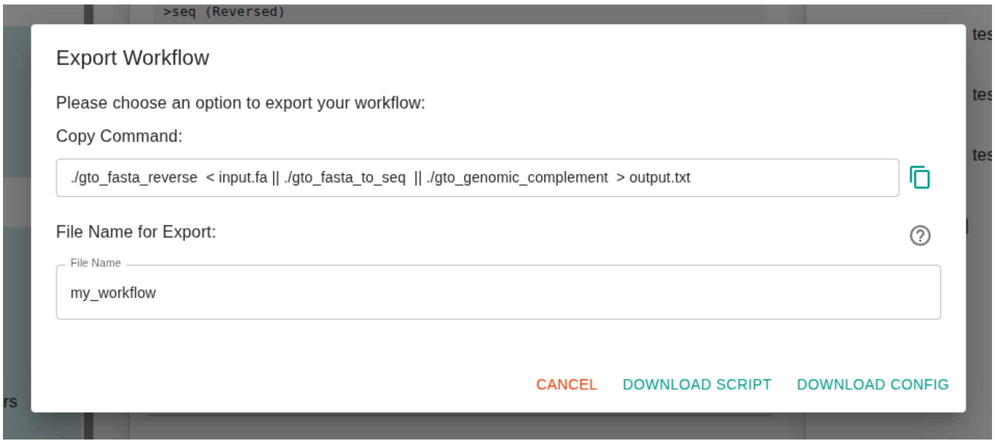

Figure 19: Export workflow modal displaying multiple export options including command line copy, script generation, and configuration file creation.

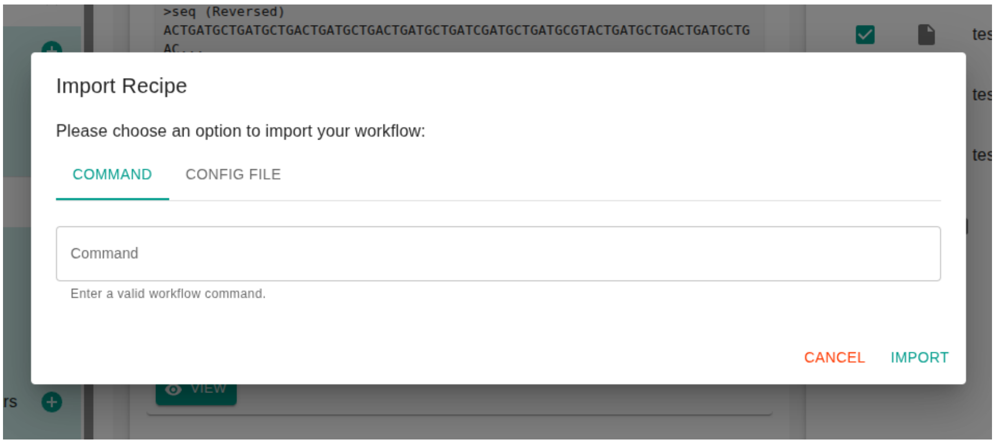

Figure 20: Import workflow modal providing options for command line and configuration file import methods.

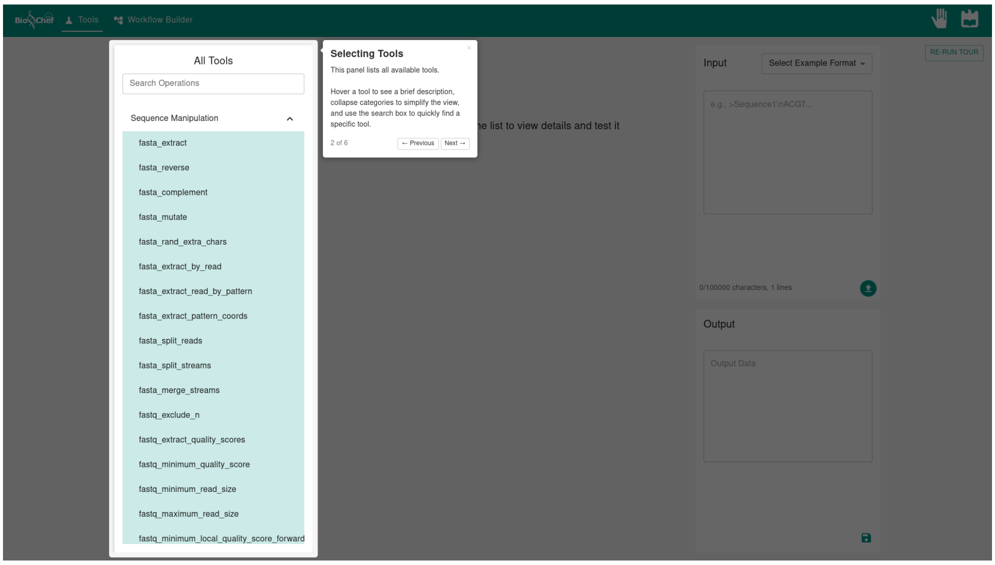

Figure 21: Interactive guided tour of the Tools page, providing step-by-step instructions for the main interface components and their functionality.

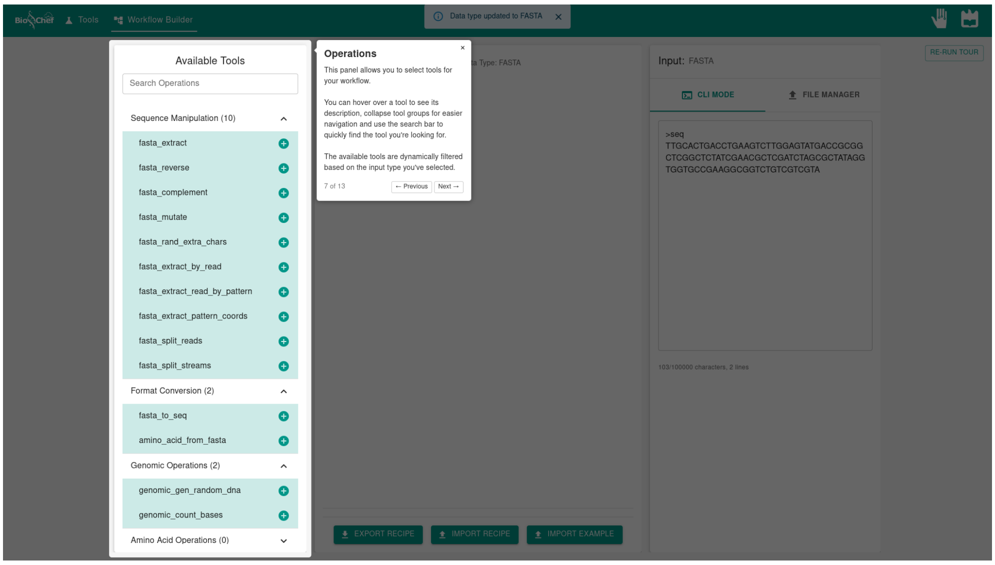

Figure 22: Interactive guided tour of the Workflows page, providing step-by-step instructions for constructing and managing workflows within the interface.
